# Recent progress in the synthesis of novel two-dimensional van der Waals materials

**DOI:** 10.1093/nsr/nwab164

**Published:** 2021-09-07

**Authors:** Renji Bian, Changcun Li, Qing Liu, Guiming Cao, Qundong Fu, Peng Meng, Jiadong Zhou, Fucai Liu, Zheng Liu

**Affiliations:** School of Optoelectronic Science and Engineering, University of Electronic Science and Technology of China, Chengdu 610054, China; Yangtze Delta Region Institute (Huzhou), University of Electronic Science and Technology of China, Huzhou 313099, China; School of Optoelectronic Science and Engineering, University of Electronic Science and Technology of China, Chengdu 610054, China; School of Optoelectronic Science and Engineering, University of Electronic Science and Technology of China, Chengdu 610054, China; School of Optoelectronic Science and Engineering, University of Electronic Science and Technology of China, Chengdu 610054, China; School of Materials Science and Engineering, Nanyang Technological University, Singapore 639798, Singapore; CNRS -International-NTU-Thales Research Alliance (CINTRA), Singapore 637553, Singapore; School of Optoelectronic Science and Engineering, University of Electronic Science and Technology of China, Chengdu 610054, China; School of Materials Science and Engineering, Nanyang Technological University, Singapore 639798, Singapore; Key Lab of Advanced Optoelectronic Quantum Architecture and Measurement (Ministry of Education), Beijing Key Lab of Nanophotonics and Ultrafine Optoelectronic Systems, and School of Physics, Beijing Institute of Technology, Beijing 100081, China; School of Optoelectronic Science and Engineering, University of Electronic Science and Technology of China, Chengdu 610054, China; Yangtze Delta Region Institute (Huzhou), University of Electronic Science and Technology of China, Huzhou 313099, China; School of Materials Science and Engineering, Nanyang Technological University, Singapore 639798, Singapore; CNRS -International-NTU-Thales Research Alliance (CINTRA), Singapore 637553, Singapore; School of Electrical and Electronic Engineering, Nanyang Technological University, Singapore 639798, Singapore

**Keywords:** two-dimensional materials, synthesis, exfoliation, chemical vapor deposition

## Abstract

The last decade has witnessed the significant progress of physical fundamental research and great success of practical application in two-dimensional (2D) van der Waals (vdW) materials since the discovery of graphene in 2004. To date, vdW materials is still a vibrant and fast-expanding field, where tremendous reports have been published covering topics from cutting-edge quantum technology to urgent green energy, and so on. Here, we briefly review the emerging hot physical topics and intriguing materials, such as 2D topological materials, piezoelectric materials, ferroelectric materials, magnetic materials and twistronic heterostructures. Then, various vdW material synthetic strategies are discussed in detail, concerning the growth mechanisms, preparation conditions and typical examples. Finally, prospects and further opportunities in the booming field of 2D materials are addressed.

## Teaser

This review article gives a brief overview of emerging 2D materials with splendid physical properties and recent research advances in synthesizing large-area 2D materials.

## INTRODUCTION

**Figure 1.**
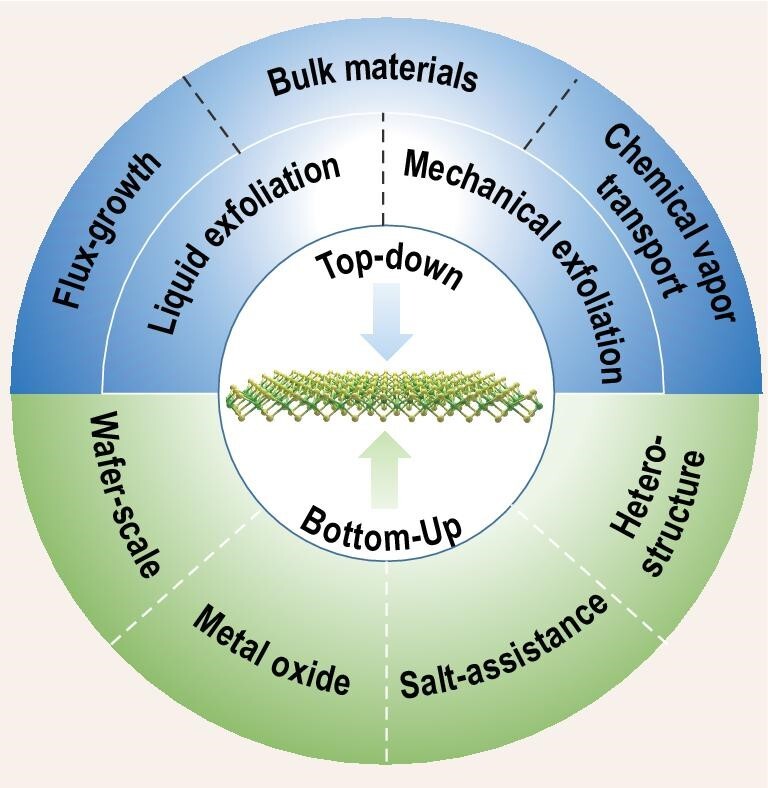
Overview of two-dimensional materials synthesis.

Since the first isolation of graphene in 2004, two-dimensional (2D) materials have received extensive attention due to their intriguing physical properties. 2D materials are bonded by strong intralayer covalent bonds and integrated by weak interlayer van der Waals (vdW) forces. Due to the antistrophic bonding strength, 2D material can be cleaved layer by layer and retain its structural integrity down to atomic thickness, which provides a novel platform for investigating low-dimensional physics and fabricating heterostructures for various applications, such as field-effect transistors (FETs) [1,2], optoelectronics [3] and sensors [4]. Recently, novel phenomena have been reported in 2D materials, such as ferroelectricity, piezoelectricity and magnetism, which are traditionally only realized in bulk material or deposited film. Meanwhile, many inspiring physical quantum phenomena, such as superconductivity, topologically protected surface states and anomalous quantum Hall effect, have also emerged, laying the foundation and groundwork for future quantum technologies [5–8]. Due to the dangling bond-free surfaces, different 2D flakes can be conveniently stacked together to fabricate heterostructures or twisted moiré patterns, providing a powerful tool for achieving atomic interface design, energy band engineering and crystal symmetry regulation. However, the challenges are that all the above physical properties rely on high-quality materials, and the compatibility with integrated circuits and future commercialization requires large-scale synthetic techniques [9,10]. To address these challenges, continuous effort has been devoted to exploring and investigating diverse synthetic methods of 2D materials. Basically, the synthetic strategies can be divided into two classes, i.e. top-down methods and bottom-up methods. In top-down methods, high-quality bulk crystals are first obtained by chemical vapor transport (CVT) [11] or flux growth method [12,13] and then the crystals are isolated into atomically thin flakes via various exfoliation technologies. Contrastingly, in bottom-up methods, vdW atomically thin flakes are prepared by assembling atoms on substrates directly, such as chemical vapor deposition (CVD), molecular beam epitaxy (MBE) or physical vapor deposition (PVD). In both bottom-up and top-down methods, different flakes can be further stacked together [8,14,15], which provide an intriguing platform for achieving various heterostructures. In very recent years, great progress has been made in the field of synthesizing large-scale and high-quality 2D materials. In this work, we firstly provide a brief overview of some emerging 2D material systems, such as topological insulators/superconductors, piezoelectrics, ferroelectrics, magnetics and twisted-angle structures with splendid physical properties. Then this paper focuses on various strategies for the preparation of 2D materials (as shown in Fig. 1). With regard to top-down methods, the mechanism and feasibility of synthetic methods for bulk vdW crystals are elaborated. The advantages and mechanisms of various exfoliation methods are discussed in detail, especially for two of the most promising exfoliation methods: electrochemical intercalation exfoliation and assisted mechanical exfoliation. With regard to bottom-up methods, the mechanism of nucleation growth in the case of using metal oxide as precursor, and the role of salt in salt-assisted CVD, are addressed. Meanwhile, the mechanism and experimental progress in growing wafer-scale 2D materials, such as hexagonal boron nitride (h-BN), MoS_2_ and WSe_2_, are discussed. Finally, prospects and further opportunities in the booming field of 2D materials are also addressed.

## EMERGING 2D MATERIALS

Recently, a large number of intriguing vdW materials were discovered. Several representative material systems, including 2D topological materials, piezoelectrics, ferroelectrics, magnetics and twisted heterostructures are introduced in the following sections.

### 2D topological materials

**Figure 2.**
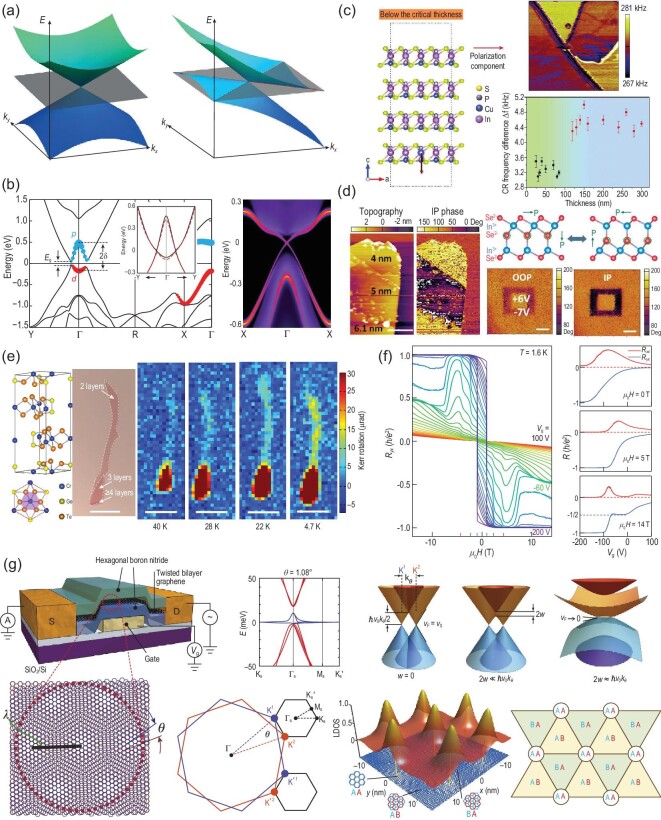
(a) Illustration of type-I electronic band dispersion (left) and type-II electronic band dispersion (right) [16]. Copyright 2015, Springer Nature. (b) Calculated electronic structures of 1T’-MX_2_: band structure of 1T’-MoS_2_ (left) and edge density of states at Γ point as a function of distance away from the edge [17]. Copyright 2014, American Association for the Advancement of Science. (c) Crystal structure, contact resonance frequency mapping and contact resonance frequency difference with various thicknesses of CuInP_2_S_6_ [33]. Copyright 2019, WILEY‐VCH Verlag GmbH & Co. KGaA, Weinheim. (d) Crystal structure and PFM investigation of α-In_2_Se_3_ (scale bars: 1 μm) [38]. Copyright 2018, American Chemical Society. (e) Crystal structure and the emergence of Kerr rotation signals of Cr_2_Ge_2_Te_6_ under 0.075 T, as the temperature decreases from 40 K to 4.7 K (scale bars: 10 μm) [41]. Copyright 2017, Springer Nature. (f) Gated-tuned QAH effect in a five-layer MnBi_2_Te_4_ flake [5]. Copyright 2020, American Association for the Advancement of Science. (g) Electronic band structure of twisted bilayer graphene (TBG) as a function of twist angle and the moiré pattern as seen in TBG [51]. Copyright 2018, Springer Nature.

In recent years, topological materials, in which the topology of band structures induces unconventional surface states and electromagnetism, have attracted broad interest in condensed matter physics. Topological materials comprise topological insulators (TIs), Weyl semimetals and topological superconductors. Due to the inverted bulk band structure, TIs are insulating in the bulk but can conduct along their surfaces. Similar to the metallic surface states in TIs, exotic topological surface states are also observed in some semimetals, called Weyl semimetals, which exhibit a cone-type dispersion around nodes (Weyl points) as shown in Fig. 2a [16]. More recently, certain 2D materials, such as transitional metal dichalcogenides (TMDCs) under appropriate circumstances, were experimentally verified as quantum spin Hall (QSH) insulators and type-II Weyl semimetals, which built a bridge between the two important fields of 2D materials and topological materials, unprecedentedly. Given the facilitation of interface engineering through forming vdW heterostructures, the 2D layered topological materials provide an excellent platform to investigate the topological properties of quantum materials. In addition, making use of the unique and exotic topological features of 2D layered topological materials, such as the protected surface states, chiral carriers of Weyl semimetals and strong spin-orbital coupling, can further promote the development of high-performance functional quantum devices.

The most representative topological systems in 2D materials are TMDCs with the chemical formula MX_2_ (M = Mo, W; X = S, Se and Te), some of which exhibit strong spin-orbit coupling and diverse crystal structures, e.g. hexagonal (2H), trigonal (1T), distorted trigonal (1T^′^) and tetragonal (T_d_) structure. Early in 2014, the 1T^′^-MX_2_ (M = W and Mo; X = S, Se and Te) monolayers with distorted trigonal structure were predicted to be endowed with QSH effect. As shown in Fig. 2b, in addition to a sizeable gap opening near the Fermi level, the band inversion of the 1T^′^-MX_2_ monolayer with distorted structure is also observed, leading to the expectation that the 1T^′^-MX_2_ monolayer would be confirmed as a 2D topological insulator. The non-trivial band topology in the 1T^′^-MX_2_ monolayer could further be modulated by external excitations, including strains and external electric fields. For example, Qian *et al.* recently reported a topological FET based on the vdW heterostructures of 2D h-BN dielectric layers, where the topological phase transition of 1T^′^-MX_2_ was exactly regulated by the electric field rather than carrier depletion [17]. In fact, the existence of a type-II Weyl fermion featured with a strongly titled Dirac cone along a certain momentum direction was first predicted in WTe_2_ and MoTe_2_. Tremendous experimental effort was devoted to the verification of the type-II Weyl fermion in both MoTe_2_ and WTe_2_ [18]. Additionally, the alloyed W_x_Mo_1−x_Te_2_ was also verified to be a type-II Weyl fermion, and the topological strength of this alloy can be continuously modulated through controlling Mo concentration [19]. Following the discovery of type-II Weyl semimetals in MoTe_2_, Zhou *et al.* proposed the existence of type-II 3D Dirac fermions in PtTe_2_ [20]. With a similar crystal lattice parameter and analogous band structure, PtSe_2_ and PdTe_2_ were also then theoretically and experimentally verified as type-II Dirac semimetals. It is worth noting that a crossover from the Dirac semimetal in 3D to the semiconductor at the 2D limit could occur when the thickness of the 3D Dirac semimetal PtSe_2_ is reduced. Intriguingly, the monolayer of PtSe_2_ exhibited a helical spin texture with spin-layer locking induced by local Rashba (R-2) effect.

The 2D topological superconductor is currently a hot topic, as they are not only involved with exotic quantum physics but also suggested to be the building blocks for topological quantum computing. Recently, the 2D monolayer W_2_N_3_ has been theoretically predicted to possess superconductivity and non-trivial topology simultaneously, which contribute to the achievement of topological superconductivity [21]. It is proposed that topological superconductivity may arise from the topological boundary states of heterostructures formed by combining superconductors and topological materials due to the proximity effect, though the successful observation of topological superconductivity can highly rely on the interface conditions. It should be noted that the edge modes of the layered heterostructures are well defined, which can be easily identified. External control of 2D topological superconductivity might be achieved as these edge modes can be readily accessed by various types of external stimuli, including an electric field, magnetic field, light and mechanical strain. The TMDCs, such as NbSe_2_, TaS_2_ and TiSe_2_, are counted as archetypal exfoliated 2D superconductors, the Curie temperature (*T_c_*) of which can be modulated via controlling the thickness [22]. Furthermore, combined with the recently discovered ferromagnetic (FM) transition metal trihalides, these 2D superconductors can play an important role in realizing 2D topological superconductivity. Nowadays, novel findings have emerged in 2D topological materials. However, this field is still in its early stage and there exist many interesting topics that remained to be explored.

### 2D piezoelectric and ferroelectric materials

2D piezoelectric materials are a subset of non-centrosymmetric materials that possess electric polarization once an external mechanical stress is applied [23], exhibiting great potential in the non-volatile memories, FET, smart robotics, force sensors, self-adaptive nanoelectronics/optoelectronics, etc. Up to now, plenty of 2D piezoelectric and ferroelectrics, such as TMDCs, CuInP_2_S_6_, α-In_2_Se_3_ and group IV monochalcogenides (general structure MX, M = Ge, Sn; X = S, Se, Te), have been experimentally reported in recent years, suggesting a thriving situation [24,25]. The early studied 2D piezoelectric material is h-BN. However, it is not paid enough attention because the insulating property of h-BN is not suitable for piezotronics. Later, monolayer TMDCs without inversion symmetry in their crystal structure were considered as the potential piezoelectric. In 2014, Wu *et al.* [26] experimentally proved the piezoelectricity in MoS_2_ flakes for the first time. The piezoelectric polarization charges are caused by the relative displacement between Mo and S ions when the structure is mechanically deformed. Almost at the same time, Zhu *et al.* [27] quantified the piezoelectric coefficient of MoS_2_ through combining a laterally applied electric field and nanoindentation. The inner carrier generation, separation, diffusion and combination can be controlled by the piezoelectric polarized charges, triggering a series of correlated interesting applications in energy harvesting, stress sensors, actuators, strain-tuned electronics and optoelectronics. Inspired by these works, the study of piezoelectricity in 2D materials began to receive due attention, and the exploration of novel piezoelectric monolayers, including InSe, GaSe, α-In_2_Se_3_, GeSe and GeS, sprung up [28].

Differently to piezoelectric materials, 2D ferroelectric materials possess remanent spontaneous polarization even after the external stimulus electric field is removed, and the polarization reversal can be achieved by applying a large enough electric field. Therefore, they can be used as the ideal candidate for high storage density, low-power consumption non-volatile random-access memories, ferroelectric capacitors, switchable diodes (polarization modulated interface barrier), ferroelectric tunnel junctions and ferroelectric FET, by utilizing the remanent polarization states and feasible switching behavior under an external electric field, and have attracted considerable attention and provoked the search for 2D ferroelectrics [29–31]. CuInP_2_S_6_, as one of the promising candidates for room-temperature interlayer 2D ferroelectricity in Fig. 2c, displays a stable out-of-plane (OOP) ferroelectric polarization [32,33]. This spontaneous polarization in CuInP_2_S_6_ is attributed to the off-centering of copper sublattices from centrosymmetric positions with respect to indium sublattices. Interestingly, CuInP_2_S_6_ still displays a stable OOP polarization even when the thickness is towards the 2D limit of 4 nm. The anti-alignment dipole in CuInP_2_S_6_ largely reduces the depolarization field inside CuInP_2_S_6_, which helps stabilize the polarization in an ultrathin 2D material. The *T_c_* values of CuInP_2_S_6_ with different thicknesses are generally an intrinsic property because the interface bonding between CuInP_2_S_6_ and the substrate is very weak. Recently, the giant negative piezoresponse and quadruple-well potential were also discovered in the CuInP_2_S_6_ multilayer by You *et al.* [34] and Brehm *et al.* [35], respectively. Two equivalent metastable states, in which some Cu atoms penetrate into the vdW gaps, are also observed showing high tolerance for the displacement of the Cu atoms in the OOP direction. In addition to the above-mentioned ferroelectric with single OOP polarization, as shown in Fig. 2d, α-In_2_Se_3_ as an emerging star ferroelectric material that possess both OOP and in-plane (IP) polarization has quickly become the focus in ferroelectric research. In 2017, Ding *et al.* [36] predicted that the ground state structure of the intrinsic prototypical α-In_2_Se_3_ quintuple layer possess both spontaneous OOP and IP electric polarization due to the lowest energy with a variant symmetry breaking Zincblende/Wurtzite crystal structure. The polarization switching could be achieved by laterally shifting the central Se layer with a modest electric field through readily accessible kinetic pathways. Zhou *et al.* [37] proved OOP piezoelectricity and ferroelectricity in 10 nm α-In_2_Se_3_ flakes by experiment in the same year, in which the ferroelectric phase contrast and domain walls boundary were clearly observed using piezoresponse force microscopy (PFM). Immediately afterwards, Cui *et al.* [38] reported layer-dependent interlayer ferroelectricity in ultrathin layered α-In_2_Se_3_, and demonstrated that α-In_2_Se_3_ exhibits intrinsically intercorrelated OOP and IP polarizations. Recently, piezoelectricity with different layer thicknesses and antiparallel alignment of the interlayer polarization have also been systematically observed in α-In_2_Se_3_ [39,40]. This promotes the understanding of ferroelectricity in 2D materials.

### 2D magnetism

2D magnetism is one of the most important properties of matter, enjoying an envious position in the areas of low-power consumption data storage, electronics and biomedicine. 2D intrinsic magnetic materials, including metals, semiconductors and magnetic topological insulators, have been extensively investigated both in theory and experiment, since the discovery of magnetism in few- and mono-layer CrI_3_ and Cr_2_Ge_2_Te_6_ in 2017 [7,41]. Cr_2_Ge_2_Te_6_ is a typical long-range ordered FM material with small magnetic anisotropy from the distorted honeycomb lattice and spin-orbit coupling from the Cr atom, as shown in Fig. 2e [41]. The identifications of thin flakes with various thicknesses and the effect of electrostatic gating have been explored, and a bipolar tunable magnetization behavior was observed by electron/hole doping [42]. Ferromagnetism of CrI_3_ flakes persisting down to monolayer was recorded by Xu *et al.* [7]. The layer-dependent polar magneto-optical Kerr effect (MOKE) measurements of CrI_3_ demonstrated that monolayer and tri-layer samples exhibit FM behavior, whereas bilayer CrI_3_ displays an antiferromagnetic (AFM) behavior because the adjacent two layers have opposite magnetic orientation and nearly compensate each other. Subsequently, the AFM coupling mechanism of bilayer CrI_3_ was probed through dual-gated and second harmonic generation methods. Interestingly, the magnetic states of few-layer CrI_3_ switch with external magnetic field, further influencing the tunneling currents. Based on this principle, the tunneling magnetoresistances (MRs) with stepped multiple configurations were realized in the tunnel junctions where bilayer to four-layer CrI_3_ flakes were used as the spin-filter tunnel barriers [43–45], achieving a record 19 000% MR ratio. These results indicate that CrI_3_ shows great potential in developing high-density, low-consumption and non-volatile memory devices.

In addition, the recently discovered intrinsic magnetic topological insulators of (MnBi(Sb)_2_Te_4_)_m_(Bi(Sb)_2_Te_3_)_n_ with spontaneously broken time-reversal symmetry create an emerging field for the realization of the quantum anomalous Hall (QAH) effect, axion insulator states and the topological magnetoelectric effect. They exhibit the controllable magnetic properties and anomalous Hall effect illustrated in Fig. 2f [5,6]. A zero-field QAH effect was observed in a five-septuple-layer flake, and the external magnetic field further increased the quantization temperature [5], which established an ideal arena for exploring various topological phenomena. In short, the advance of 2D magnetic materials provides a platform for exploring the underlying physics of magnetism and demonstrates the feasibility of emerging applications.

### Twisted heterostructure

Constructing heterostructures is a common and popular approach for investigating and modulating the properties of 2D materials. The vdW heterostructures can be created simply by mechanically stacking different monolayers together along the vertical or lateral direction. As the 2D monolayer materials intrinsically exhibit extraordinary properties, pioneering strategies, such as external excitation, stacking sequence and crystallographic alignment, can be implemented to explore the potential fascinating properties in vdW heterostructures. More intriguingly, vdW heterostructures can provide a material platform for the emergence of interlayer excitons. Furthermore, manipulation of these interlayer excitons even enables the development of excitonic integrated circuits, especially in optical communication and signal processing [46]. Recent years have witnessed the achievement of TMDC-based vdW heterostructures with type-II band alignment, where the conduction band minimum (CBM) and valence band maximum (VBM) are located in different monolayers, facilitating the formation of interlayer excitons. Electrons tend to accumulate in the layer with the lower CBM while holes incline to accumulate in the other layer with the higher VBM. Meanwhile, strong electron-hole Coulomb interactions can be formed since the interlayer separation is limited to almost atomic-layer thickness, which guarantees the spatially separated but bounded electron-hole pairs [47]. Due to the spatially indirect nature of the vdW heterostructures, excitons were expected to have a long lifetime and this is exactly what was observed in these bounded electron-hole pairs [48]. Due to the formed OOP electrical dipole moment, the spatially indirect feature also creates the possibility of electrically controlling the optical and transport properties of the interlayer excitons. Besides, the interlayer excitons in the heterostructures also exhibit great potential application in valleytronic devices as they inherit valley-contrasting physics from the constituent monolayers [46–48]. The atomically thin vdW heterostructures can be a treasure house for exploring novel collective quantum phenomena and the interesting new device.

Conventional vdW stacking seldom considers the aligned angle between different layers. However, emerging twistronics indicate that the aligned angle is a new dimension to design the energy band. When two flakes with the same or similar lattices are misaligned with each other in some angle, an additional superlattice, called a moiré pattern, can be formed. The periodic structure generates new energy states in the energy band. The moiré pattern or commensurate structure is highly dependent on the lattice mismatch and rotation angle between the twisted bilayers. The moiré period is given by ${\rm{b}} \approx {\rm{a}}/\sqrt {{\delta ^2} + {\theta ^2}} $, where $ \rm{a} $ is the monolayer lattice constant, *δ* is the lattice mismatch and *θ* is the relative twist angle [49]. Usually, it can vary by several nanometers to tens of nanometers as shown in Fig. 2g. Early in 2011, Bistritzer and Macdonald theoretically predicted that the moiré band near Fermi energy could be flat when *θ* approached 1.05°, though graphene does not have the intrinsic flat band structure at low energies [50]. Later, in 2018, Cao *et al.* experimentally demonstrated that twisted bilayer graphene with small twist angles (∼1.1°) presents unconventional superconducting and magneto-transport [51,52]. Recently, Cao *et al.* demonstrated that electric-field-tunable superconductivity could even be achieved in alternating-twist magic-angle tri-layer graphene [53]. Twisted bilayer graphene with flat bands has attracted much attention as it provides a new excellent material platform for exploring many-body quantum phases and exotic correlated systems. Apart from twisted bilayer graphene with a magic angle, twisted multilayer graphene with a large twisted angle of 30°, due to the interlayer coupling induced by the moiré potential, also exhibits novel phenomena [54]. Researchers have also fabricated graphene/h-BN and graphene/black phosphorus heterostructures with moiré superlattices by utilizing the near-lattice matching of two crystals.

The emerging pseudo-Landau levels are considered to affect the electric properties of these heterostructures. For example, in TMDCs and TMDC-based heterostructures, the emerged moiré superlattices bring about modified properties and novel characteristics, including moiré phonons, moiré excitons, band structure reconstruction and other topological transitions. These exotic characteristics are involved in the direct modulation of momentum space by reciprocal lattices of moiré superlattices and the alterations of phonon modes and charge distribution due to the corresponding interlayer interactions. For example, the twist-angle-dependent photoluminescence (PL) emission behaviors in the twisted bilayer TMDCs hugely rely on the properties of new excitons in the moiré superlattice. In summary, these novel phenomena are highly associated with interlayer interactions and the reciprocal lattices of moiré superlattices.

## TOP-DOWN GROWTH METHOD

The synthesis of 2D materials has been explored for decades and can be divided into two synthetic strategies, known as top-down and bottom-up. In top-down synthetic strategies, single- and few-layer vdW flakes are prepared by the dissociation of bulk crystals. Therefore, a brief overview of the growth methods for vdW bulk crystals and the process for the preparation of mono- or few-layer flakes are presented in this section.

### Methods for bulk single crystals

**Figure 3.**
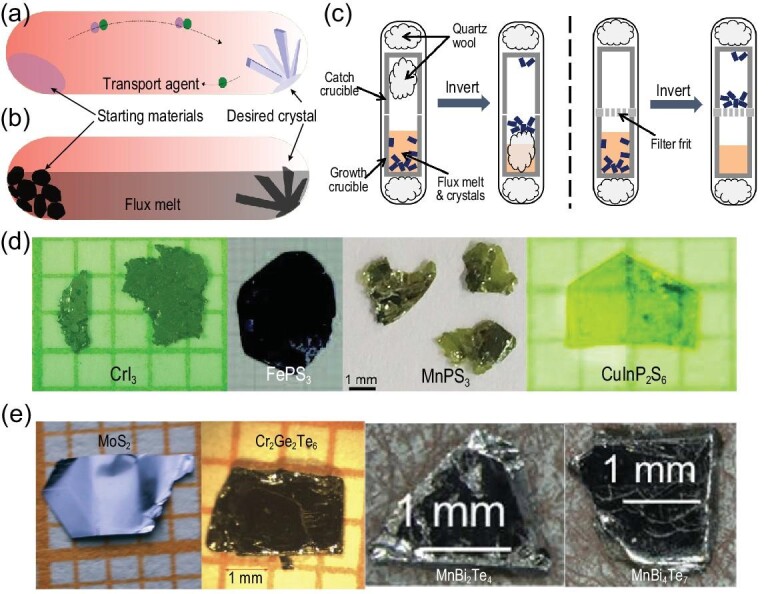
Scheme diagrams of (a) CVT, flux growth methods with (b) horizontal and (c) vertical configuration for fabrication of 2D materials [13,57]. Copyright 2017, American Physical Society; Copyright 2020, AIP Publishing. The typical single crystals obtained by (d) CVT and (e) flux growth methods [6,33,60–64]. Copyright 2019, American Association for the Advancement of Science; Copyright 2019, WILEY‐VCH Verlag GmbH & Co. KGaA, Weinheim; Copyright 2018, American Physical Society; Copyright 2019, AIP Publishing; Copyright 2016, The Japan Society of Applied Physics; Copyright 2015, The Royal Society of Chemistry; Copyright 2019, Chinese Physical Society and IOP Publishing Ltd.

The growth methods for a bulk vdW single crystal continue to receive much attention because single crystals are one of the most important sources of high-quality mono- or few-layers. The methods can be classified into two main categories: CVT and flux methods. As an old yet powerful technology, CVT reaction plays an important role in the growth of bulk vdW materials. Schafer carried out systematic research and description of CVT reactions in the 1950s and 1960s. Since then, a significant proportion of emerging vdW materials have been fabricated through CVT reaction, including TMDCs, halides (such as CrI_3_, CrBr_3_), TMPX_3_ (such as FePS_3_, MnPS_3_, CoPS_3_, NiPS_3_, CuInP_2_S_6_) and Fe_3_GeTe_2_. However, it is very difficult to obtain the desired crystals in systems where strongly non-congruent vapor phases during the transport process are usually generated [55]. The mechanisms of a vast number of CVT reactions hardly differ, as comprehensively discussed in the book entitled *Chemical Vapor Transport Reaction* [56]. As shown in Fig. 3a, a CVT reaction in a closed system generally can be simplified into three processes: sublimation, transport and deposition. An ideal CVT reaction should present good reversibility at a certain temperature range, which means that the equilibrium constant of the reaction should be close to one. However, it is a challenge to precisely control the reaction due to the complex relationship between these influencing factors and reaction processes [11]. Thus, it is necessary to have a full understanding of the corresponding reaction details and formation mechanisms to produce high-quality crystals and explore 2D materials with new properties and structures.

Flux growth, as another important growing method for 2D materials, utilizes a high-temperature melt of inorganic compounds as the solvent for crystallization [12]. The flux, which is usually simple inorganic solids at room temperature, such as KCl, NaCl, CsCl, AlCl_3_, Te, Sn and Bi, melts at a conveniently low temperature. Sometimes, combinations of several inorganic compounds are used to form an even lower melting eutectic liquid. A flux growth with horizontal configuration, as shown in Fig. 3b, is also considered as liquid transport growth, where the flux does not substantially generate with the target product during crystal growth but acts as a liquid transport agent. The flux is melted into a liquid and dissolves the source materials at high temperature. Driven by the composition gradient, the dissolved source materials are transferred to the cold zone. Then, the thermodynamically stable phase starts to crystallize, forming the desired single crystal at the cold zone. To make an effective transport, the inorganic compound flux should meet certain conditions as follows: (i) have the ability to dissolve a substantial quantity of the source materials, (ii) be unreactive with the quartz tube container, (iii) be easily removed after growth, (iv) have low toxicity, (v) be commercially available with low cost, (vi) have low volatility, (vii) be unreactive with the source materials yielding new impurities and (viii) have a moderate melting point. As shown in Fig. 3c, in a conventional vertical flux growth, single crystals are generally grown from eutectic liquid according to the phase diagrams [57]. When the flux is composed of one or more elements of the targeted crystals, it is also called self-flux growth. The source materials are completely dissolved into the flux forming a homogeneous eutectic liquid. The homogeneous eutectic liquid will be supersaturated, and the high-quality desired crystal will slowly nucleate and crystallize at a typical point, when the temperature of the system is slowly reduced [58,59]. Although flux growth displays great potential for the preparation of high-quality and diversified vdW crystals, it is difficult to synthesize certain complex crystals without the accurate phase diagram [60–64].

### Exfoliation of bulk material

Atomic-scale 2D flakes can be obtained by the exfoliation of their bulk materials. The exfoliation can be split into two major categories: liquid phase exfoliation and mechanical exfoliation. Great efforts have been devoted to innovating novel strategies to prepare large-area, high-quality 2D flakes in recent years.

### Liquid phase exfoliation

Liquid phase exfoliation can achieve large quantities of dispersed mono- or few-layer flakes from bulk crystals in a specific solvent. It is divided into direct exfoliation and intercalation exfoliation depending on the types of forces that facilitate exfoliation. In 2011, Coleman *et al.* [65] directly exfoliated graphite into mono- or few-layer graphene by ultrasonication in various common solvents, such as N-methyl-2-pyrrolidone (NMP) and dimethylformamide (DMF), where the organic solvents play a key role in transferring mechanical force to vdW materials during the exfoliation process. It is notable that the enthalpy of exfoliation for vdW materials is minimized in this process, because the surface tension of these solvents is close to the surface energy of vdW materials. Later, this exfoliation method was improved and expanded to produce other high-quality mono- or few-layer vdW flakes, as summarized by Nicolosi *et al.* [66] and Cai *et al.* [67]. However, with direct exfoliation it is difficult to obtain uniform atomic-scale flakes and this presents some drawbacks, such as low yields and relatively small flakes.

It is more feasible for intercalation exfoliation to produce uniform large-area atomic flakes than direct exfoliation. The familiar intercalation materials are the alkali metal atoms or ions, because they can easily enter the interlayer gap of vdW materials by treating vdW materials with a solution of alkali metal in liquid ammonia [68] or naphthalide [69], or *n*-butyllithium in hexane [70]. The hydration in liquid will induce the production of bubbles, expand the interlayer spacing and weaken the vdW force, resulting in the isolation of vdW materials in the dispersion solution. The detailed mechanism of intercalation and chemical exfoliation of alkali metals was illustrated by Cai *et al.* in 2018 [67]. In addition, other ions, atoms or molecules, including transition metal ions, anions, ammonium ions and organic molecules, can also be intercalated into vdW materials to produce flakes with atomic thickness [67,71]. However, intercalation exfoliation and direct exfoliation typically require violent, chemically and physically aggressive methods, yielding small and defective flakes that are not suitable for device fabrication.

To improve the efficiency and controllability of liquid phase exfoliation to produce large-area atomically 2D materials, electrochemical intercalation as a mild exfoliation methodology was proposed and has received intense attention in recent years [72,73]. In this process, the current acts as an attractive driving force to bring foreign molecules or ions into the bulk vdW materials, thereby expanding the interlayer spacing, and producing mono- or few-layer flakes. The rate and extent of reaction can be controlled by the current and discharge capacities, and is usually finished in several minutes to hours with obvious structural deformation and redox reactions at the electrodes. Generally, the intercalation speed is very fast, because the surface oxidation can quickly open the interlayer channels at the edges. At the same time, structural defects of flakes inevitably form during the production process.

**Figure 4.**
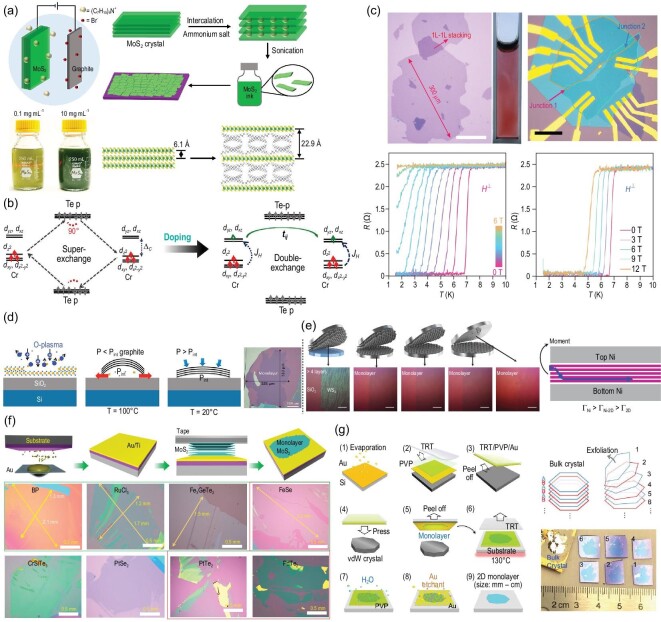
(a) Schematic of the electrochemical intercalation of MoS_2_ with tetraheptylammonium bromide, and the photograph of exfoliated MoS_2_ nanosheets dispersed in isopropanol with different concentrations [77]. Copyright 2018, Springer Nature. (b) Schematic diagram of the super exchange interaction in Cr_2_Ge_2_Te_6_ and the double-exchange interaction in (TBA)Cr_2_Ge_2_Te_6_ [75]. Copyright 2019, American Chemical Society. (c) Optical microscopic images of a representative large-sized NbSe_2_ monolayer (∼300 μm; scale bar: 100 μm) and twisted NbSe_2_ device (scale bar: 20 μm). Temperature-dependent longitudinal resistance of a printed NbSe_2_ film under different out-of-plane and in-plane magnetic fields [8]. Copyright 2020, Springer Nature. (d) Key steps of exfoliation process for 2D materials with oxygen plasma cleaning removing adsorbates from the SiO_2_/Si substrate, and the optical microscopic image of obtained graphene [81]. Copyright 2015, American Chemical Society. (e) The layer-resolved splitting process, schematics and optical micrographs for as-exfoliated thick WS_2_ and monolayers of WS_2_ (scale bars: 50 μm) [84]. Copyright 2020, American Association for the Advancement of Science. (f and g) Schematics of the exfoliation process of improved gold-assisted exfoliation and optical images of the as-exfoliated monolayer 2D flakes [14,15]. Copyright 2020, Springer Nature; Copyright 2020, American Association for the Advancement of Science.

In addition, it is found that cathodic electrochemical intercalation is conducive to expanding the interlayer spacing during the reduction reaction, and the cations are naturally intercalated into the expanded gap to form the exfoliated atomically 2D flakes [8,74–76]. Thus, it creates great potential for exfoliation of large-area high-quality emerging 2D flakes, and rapidly becomes one of the efficient methods of exfoliating emerged 2D materials [74]. In 2018, Duan’s group [77] made use of the cathodic electrochemical intercalation of quaternary ammonium molecules to synthesize MoS_2_ layers in Fig. 4a. The pure-phase semiconducting 2H-MoS_2_ thin flakes with a narrow thickness distribution were obtained by precisely controlling the exfoliation process. The obtained flakes displayed a good electrical performance in terms of mobility (10 cm^2^ V^–1^ S^–1^) and on/off radio ∼10^6^. This approach was further expanded to fabricate other 2D materials, including WSe_2_, Bi_2_Se_3_, NbSe_2_, In_2_Se_3_, Sb_2_Te_3_ and black phosphorus [78,79]. Recently, Wang and co-workers [75] applied this method to the FM system and successfully exfoliated Cr_2_Ge_2_Te_6_ crystals via intercalation of organic electrodes (tetrabutyl ammonium, TBA^+^) to prepare large-area magnetic thin flakes and manipulate their magnetic properties. In the intercalation process, Cr_2_Ge_2_Te_6_ crystals were pressed onto an indium plate and acted as the positive electrode with a silver piece as the negative electrode in tetrabutyl ammonium bromide-DMF solvents. The Cr_2_Ge_2_Te_6_ crystal obtained electrons forming (TBA^+^)_x_Cr_2_Ge_2_Te_6_ with increased interlayer spacing, promoting the exfoliation of large-area monolayer flakes. Surprisingly, the formed (TBA^+^)_x_Cr_2_Ge_2_Te_6_ flakes displayed a typically metallic behavior at low temperature due to electron doping. Further, their underlying mechanism of ferromagnetism changed to metallic double-exchange from weak super-exchange of bulk materials, as displayed in Fig. 4b. This change also resulted in a significantly increased *T_c_* value from 67 K to 208 K, and a drastic change of magnetic easy-axis from the *c*-axis for Cr_2_Ge_2_Te_6_ to *ab*-plane for (TBA^+^)_x_Cr_2_Ge_2_Te_6_, which provided a novel approach to manipulating the magnetic and electronic properties in emerging 2D materials. Similarly, Li *et al.* [8] adjusted homogeneity of the process by strictly controlling the dilute concentration of ions, causing relatively small charge transfer and weakening of vdW bonding between layers, thereby gently delaminating large flakes into the liquid. A high yield of 75% with large-size monolayers up to 300 μm was demonstrated for NbSe_2_. The produced NbSe_2_ monolayer exhibited low defect density, high single crystallinity, low residual resistivity and typical hallmarks of superconductivity, as shown in Fig. 4c. It is interesting that the absorbed solvent molecules on the surface of monolayer NbSe_2_ can form a protective layer to render it more stable in air and that they will vacate the interface via a self-cleaning effect upon stacking.

### Mechanical exfoliation

Mechanical exfoliation is a convenient method of obtaining high-quality mono- or few-layer flakes from bulk crystals, displaying a central role in the exploration of emergent vdW materials. To be specific, a piece of bulk crystal is stuck on Scotch tape, and the mono- or few-layer flakes can be obtained by repeating the peeling-off process multiple times. Various 2D flakes have been successfully obtained by this nearly unchanged mechanical exfoliation method since the discovery of graphene in 2004. The as-exfoliated flakes usually have a clean surface, could be easily transferred to various substrates, and have aroused immense interest with regard to the development of next-generation electric devices. However, the relatively low yield and small size of vdW materials severely restrict their development in both research and application. To solve these problems, various technologies assisting mechanical exfoliation were hotly explored in recent years. Moldt *et al.* [80] conducted an anodic bonding process to exfoliate graphene, substantially increasing the yield and size of graphene flakes over conventional exfoliation methods. Generally, the critical factor for exfoliating vdW materials is that the bonding strength between the substrate and the outermost flakes of materials should be larger than the vdW forces of interlayer flakes. Reinforcing the interaction between vdW materials and substrates plays a key role in producing larger-area vdW monolayer flakes. In 2015, Huang *et al.* [81] produced high-quality few-layered graphene flakes with a size of several hundred microns on SiO_2_/Si substrate using oxygen plasma cleaning, as shown in Fig. 4d. Compared with the direct mechanical exfoliation, this simple process increased the yield and the area of the transferred flakes 50-fold. It can also be applied to other 2D materials, providing an effective way of producing larger-area high-quality flakes of various 2D materials, though the success rate may be drastically reduced if the interaction between vdW materials (such as TMDCs) and SiO_2_ substrate was small.

To uniformly enhance the interaction between target vdW materials and substrate, suitable mediums that firmly adhere to 2D crystals without causing their structure and properties to deteriorate are necessary. In 2010, Unarunotai *et al.* [82] employed a palladium (Pd) and polyimide layer as the medium for the preparation of monolayer graphene (Gr) and an area yield of almost 100% was achieved. The cracks at the interface of Gr/Gr were usually induced, because the adhesion energy graphene interlayer is lower than that of Gr/Pd-polymide. Taking advantage of the high binding energy between Gr and nickel (Ni), it is a promising solution to achieve crack-assisted layer transfer using Ni as the medium [83]. Subsequently, this approach was extended to exfoliate various wafer-scale 2D materials by Shim *et al.* [84]. In this process, a robust interface toughness was formed between the deposited Ni layer and vdW materials, which was three times that of the interface between vdW layers. The edge crack propagates at the interface of 2D/2D layer followed by spalling mode fracture in Fig. 4e, when a mechanical force was applied to the top Ni in an Ni layers sandwich vdW material structure. The cracks initiate from the bottom and propagate downward, resulting in a single vdW monolayer on the bottom Ni layer. Further, the heterostructures formed by this technology, together with quasi dry stacking, exhibited a uniform performance across the entire wafer, promoting the commercialization of vdW flake-based devices. However, parasitic crack propagation is frequently observed in the process of subsurface cracking, resulting in high surface roughness and undesired surface structure [85].

Compared with crack-assisted exfoliation, gold-assisted exfoliation of 2D materials can effectively avoid this problem. Gold displays a moderate affinity to form semi-covalent bonds with most of vdW materials terminated by chalcogens and halogens elements. It also displays low chemical reactivity and high air stability, and appears to be a promising candidate for high-yield and large-area exfoliation of 2D materials [14,86]. In 2016, Desai *et al.* [87] used gold-assisted exfoliation for the first time to produce TMDC monolayers up to 500 μm in lateral dimensions from their bulk crystal. The evaporated gold film on the topmost layer of the bulk TMDC crystals selectively increased the adhesion, and the topmost layer can be completely peeled off from the bulk crystal. The gold-assisted exfoliated MoS_2_ monolayers displayed almost 100% internal quantum yield at low pump power, and the extracted mobility values were consistent with the value ranges observed in traditional mechanical exfoliated samples, which advances the possibility of large-scale fabrication of monolayer TMDC devices. However, this direct deposition of metal on a TMDC bulk crystal will introduce considerable defects in a TMDC monolayer. In order to avoid this drawback, Velicky *et al.* [88] improved the gold-assisted exfoliation method in 2018, in which the freshly cleaved bulk crystal on tape was brought in contact with the gold film deposited onto a substrate, as shown in Fig. 4f. The size of the produced MoS_2_ monolayers can reach centimeter scale, which is almost the same as the size of the bulk crystal. The strong interaction between gold and the topmost MoS_2_ layer was confirmed by spectroscopic, microscopic and first-principles density functional theory (DFT) analyses. It is worth mentioning that a strong interaction did not appear in all the gold film and MoS_2_ interfaces. The gold films exposed to air for >15 minutes will cause a non-negligible effect for 2D materials. The exposed gold film adsorbed the organic airborne contaminants in air, resulting in a transformation of gold surface from hydrophilic to hydrophobic, which weakened the adhesion forces between the gold and the topmost layer of bulk crystals. Subsequently, Huang *et al.* [14] expanded the scope of gold-assisted exfoliation to produce large-area vdW monolayers. The interlayer binding energies of a large number of vdW crystals and their adhesion energy to gold surfaces were evaluated using DFT calculation, indicating that gold interacts strongly with the terminate elements (chalcogens and halogens) in most 2D materials. So far, around 40 high-quality monolayered 2D materials with macroscopic size were demonstrated, including topological, ferroelectric and FM. Using a similar method in Fig. 4g, Liu *et al.* [15] disassembled some TMDC materials into high-quality microscopic monolayers and reassembled them into an artificial structure with controllable properties, such as hetero-bilayers with non-linear optical properties and controlled twist angle, which is closer to the mass production of macroscopic monolayers and commercial preparation. Advanced metal-assisted mechanical exfoliation may stimulate a new research upsurge of 2D materials in the future.

## BOTTOM-UP GROWTH METHODS

To efficiently synthesize large-area 2D material films, CVD, as a promising candidate in bottom-up methods, is introduced and developed. Early in 2009, uniform large-area graphene film was first obtained on Cu foils by the CVD method [89]. After that, the last few decades have witnessed its success on numerous 2D materials, such as MoS_2_ [90–93], WS_2_ [94,95], ReS_2_ [96] and corresponding vdW heterostructures [97,98]. As a powerful tool, CVD can also be used to synthesize 2D non-layered compounds, such as transition metal carbides and nitrides. However, these non-layered compounds with unsaturated surface dangling bonds can be oxidized when exposed to air. Recently, Ren *et al.* [99] synthesized a novel 2D material MoSi_2_N_4_ without known 3D parents, in which the middle layer MoN_2_ was sandwiched by two layers of SiN. Only micrometer-scale non-layered 2D Mo_2_N domains around 10 nm thick were obtained without adding elemental Si. In contrast, the new 2D MoSi_2_N_4_ was created when the raw materials contained the elemental Si during CVD growth. They proposed a general route to prepare such materials using proper elements to passivate the high-energy surfaces of non-layered materials during growth. Another 2D materials monolayer WSi_2_N_4_ was synthesized successfully using the same approach [100]. Nevertheless, we mainly focus on 2D layered materials in this part. In order to realize the full benefits of 2D layered materials in electronic and optoelectronic devices, clean surfaces, few defects and larger domain size of 2D materials can be a prerequisite. The mechanism of CVD growth turns out to be a significantly important guideline for the synthesis of high-quality 2D materials. Thus, in this section, we will discuss the basic principle of CVD growth in a single-crystal wafer-scale film and vertical heterostructures.

### Mechanism of metal oxide nucleation growth

**Figure 5.**
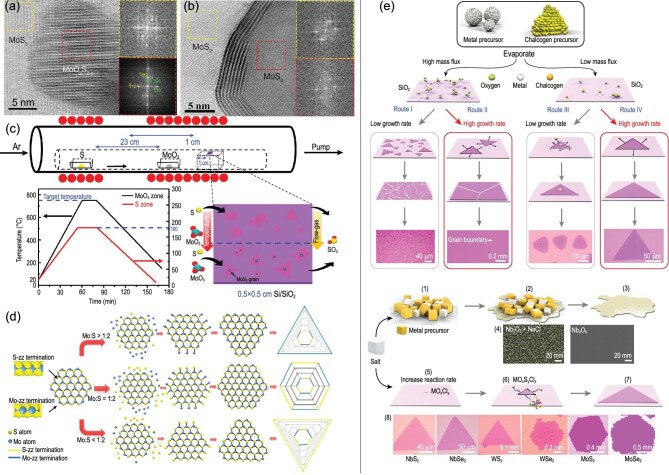
Bright field-scanning transmission electron microscopy image of the structure of (a) the empty area of the core and (b) nanoparticles without the core-shell fullerene structure, and the corresponding fast Fourier transform patterns gathered from selected regions on few-layer MoS_2_ (yellow dotted circle) and MoO_x_S_2−y_ nanoparticle (red dotted circle) [102]. Copyright 2017, Springer Nature. (c) The temperament curve of CVD reaction furnace and inset is a schematic view of SiO_2_/Si substrate illustrating the growth of MoS_2_ under different temperatures and gas fluxes, and temperature evolution of the reaction furnace. The black and red curves indicate the temperature evolution of central heating zone and S zone, respectively [107]. Copyright 2017, American Chemical Society. (d) The schematic diagram illustrates the domain shape changing procedure dependent on the growing rates of two different terminations [108]. Copyright 2014, American Chemical Society. (e) Metal oxychlorides are formed, and these promote the reactions and flow chart of the general growth process for the production of TMDCs by the CVD method [112]. Copyright 2018, Springer Nature.

In general, there are three stages in the growth process of conventional CVD. In the first stage (transportation), the solid precursors are sublimates at high temperature and then transported to the specific growth area by the carrier gas. In the second stage (nucleation), the gaseous precursors diffuse to the substrate, forming the nucleus. In the third stage (growth), the gaseous precursors continually react and then aggregate near the nucleus, thereby realizing the epitaxial growth of the target product. The nucleation mechanism in CVD is rather controversial, and there exist two popular perspectives: (i) the metal oxides react with the chalcogenides, forming intermediate volatile metal oxi-chalcogenides, and then the volatile metal oxi-chalcogenides further react with chalcogenides to evolve into fully 2D MX_2_ [101,102]; (ii) the metal oxides and chalcogenides react completely in vapor phase and the obtained MX_2_ is subsequently deposited on the desired substrate [103]. Zhu *et al.* [102] directly observed two types of seeding centers via transmission electron microscopy: (i) Mo-oxysulfide nanoparticles either in nested multi-shelled fullerene-like structures or compact nanocrystals as demonstrated in Fig. 5a, and (ii) atomic-scale MoS_2_ monolayer clusters as shown in Fig. 5b. Most recently, a new self-seeding mechanism was proposed in the growth of MoS_2_, WS_2_, MoSe_2_ and their heterostructures [104–106]. Liang *et al.* [104] reported the chemical evolution pictures from 0D MoO_3-x_ nanoparticles to 2D MoS_2_ layer. In the beginning, volatile MoO_3−x_ were generated from the reduced MoO_3_ particles in sulfur steam. Most of these volatile MoO_3−x_ escaped from the substrate during warming, while residual MoO_3−x_ aggregated together, forming small and stable MoO_3−x_ particles. Then, the MoO_3−x_ particles would be further reduced by sulfur vapor to form MoS_2_ particles. Finally, the 2D MoS_2_ layer growth started from the edge of the nanoparticles. Cain *et al.* [105] pointed out that the nucleation sites of suboxide nanoparticles originated from the ‘self-seeding’ and were further reduced and formed the monolayer. As a result, the orthorhombic crystal structure of suboxide or oxi-chalcogenide usually occurs in a sulfur/selenium-poor atmosphere while TMDC fullerenes are easily formed in a sulfur/selenium-rich atmosphere. A moderately reducing environment is available for the formation of a TMDC monolayer. Moreover, the two different nucleation mechanisms (i.e. 2D planar nucleation and self-seeding nucleation) [107] could interconvert into each other through tuning the growth temperature, flux of carrier gas and the concentration of precursors on SiO_2_/Si substrate, as shown in Fig. 5c. For example, a relatively lower precursor concentration facilitates 2D planar nucleation, which is responsible for the formation of monolayer and bilayer MoS_2_. In contrast, a higher precursor concentration induces a self-seeding nucleation mechanism, which easily produces few-layer and multilayer MoS_2_.

As for the growth mechanism, Wang *et al.* [108] explicitly explained the evolution of MoS_2_ shape, which is strongly correlated with the precursor ratio, as shown in Fig. 5d. Particularly, when the ratio of Mo and S is non-stoichiometric, the final domain shape will transform to triangular because of the different growing rates. In such a case, the only difference lies in its edge termination. When Mo is rich, the domain shape is terminated with Mo edges and vice versa. While the Mo : S ratio is stoichiometric, Mo zigzag termination and S zigzag termination had similar growing rates, which resulted in forming the hexagonal domain shape. Combining the experimental data, Rajan *et al.* [109] proposed a generalized mechanistic model to explain the evolution of MoS_2_ monolayer shape quantitatively. Yang *et al.* [110] found that the growth temperature also has significant effect on the domain shape. The growth temperature can break the dynamic balance between MoO_3−x_ and S. Three shapes, namely three-point star, triangle and hexagonal flakes, can be obtained in three different growth conditions.

### Salt-assisted CVD growth

Not all 2D materials can be synthesized using thermal CVD due to the high sublimation temperature of partial source metal oxide (i.e. WO_3_). In 2015, Li *et al.* [111] added alkali metal halides into precursors for growing WSe_2_ and WS_2_ monolayers at moderate temperatures and atmospheric pressure. Until now, the synthesis of many types of 2D materials is boosted by the salt-assisted CVD method. Zhou *et al.* [112] prepared 47 compounds and heterostructures by introducing halide-salt in a CVD system. According to the mass flux of precursor and growth rate, one specific 2D TMDC can be formed via four routes as shown in Fig. 5e. Specifically, a high-mass flux metal precursor produces large-scale continuous monolayer polycrystalline films with different domain sizes. At that time, a low growth rate contributed to the fabrication of a monolayer polycrystalline film with small grains, while high growth rate tended to form continuous monolayer films with large grains of up to millimeters in size. However, low-mass flux of a metal precursor can generate separated single-crystalline monolayers with small or large sizes depending on the growth rate. Generally, the melting points of metal elements or metal compounds can be reduced by adding suitable salts, as shown in Fig. 5e. For example, high melting point Nb_2_O_5_ usually cannot form an Nb nucleus but a high-mass flux of Nb metal can be obtained by the salt-assisted method. Besides, the salt may react with some metal-compounds to form volatile metal oxychlorides. This accelerates the reaction rate with chalcogenide. Taking the precursors of MoO_3_ and NaCl as an example, the reaction equations can be expressed as follows:

(1) \begin{eqnarray*} \!\!\!\!2{\rm{Mo}}{{\rm{O}}_3}\left( {\rm{s}} \right) + 2{\rm{NaCl}}\left( {\rm{s}} \right) &\to &{\rm{N}}{{\rm{a}}_2}{\rm{Mo}}{{\rm{O}}_4}\left( {\rm{l}} \right)\nonumber\\ &+&{\rm{Mo}}{{\rm{O}}_2}{\rm{C}}{{\rm{l}}_2}\left( {\rm{g}} \right), \end{eqnarray*}

(2) \begin{eqnarray*} 3{\rm{Mo}}{{\rm{O}}_3}\,({\rm{s}}){\rm{ }} + {\rm{ }}2{\rm{NaCl}}\,({\rm{s}}) &\to &{\rm{N}}{{\rm{a}}_2}{\rm{M}}{{\rm{o}}_2}{{\rm{O}}_7}\,({\rm{l}})\nonumber\\ &+& {\rm{Mo}}{{\rm{O}}_2}{\rm{C}}{{\rm{l}}_2}\,({\rm{g}}). \end{eqnarray*}

The thermogravimetric analysis showed that the MoO_3_-NaCl mixture largely lost major weight at ∼550°C, which was obviously lower than pure MoO_3_. The volatile product MoO_2_Cl_2_ was created via the reaction of MoO_3_ and NaCl at a temperature below the melt points of the two agents [113]. Owing to the low sublimation temperature (175°C) of MoO_2_Cl_2_, the gas precursors MoO_2_Cl_2_, S and H_2_ reacted faster and finally contributed to the nucleation and lateral growth of 2D flakes. The whole growth process only took 3 minutes and the growth rate was up to 8 μm s^−1^.

Moreover, the salt-assisted CVD method also enables growth of metal phased 2D materials. Typically, the final product is always semiconductor phase (2H) rather than metal phase (1T^′^), when synthesizing MoS_2_ through one-step thermal CVD. Recently, Liu *et al.* [114] directly prepared high phase-purity monolayer 1T^′^ MoS_2_ by choosing proper alkali-salt K_2_MoS_4_ as a precursor in the CVD system. The stability of 2H and 1T^′^ phases was controlled by the K concentration. The concentration of K was beyond 44%, and the 1T^′^ phase could be obtained on mica while the product MoS_2_ was still 2H phase at a low concentration of K. The content of K was tuned in an inert or reductive atmosphere. In an inert gas atmosphere, the formula was as follows:

$$\begin{equation*}
{{\rm{K}}_2}{\rm{Mo}}{{\rm{S}}_4} \to {\rm{Mo}}{{\rm{S}}_2} + {{\rm{K}}_2}{\rm{S}} + {\rm{S}}.
\end{equation*}$$

The K_2_MoS_4_ decomposed to pure MoS_2_ without K and formed the stable 2H phase MoS_2_. While the atmosphere was reductive, the reaction equation was as follows:

$$\begin{eqnarray*}
{{\rm{K}}_2}{\rm{Mo}}{{\rm{S}}_4} + {{\rm{H}}_2} &\to &{{\rm{K}}_{\rm{x}}}{\rm{Mo}}{{\rm{S}}_2} + {\rm{K}} + {{\rm{K}}_2}{\rm{S}} +\, {{\rm{H}}_2}{\rm{S}}.
\end{eqnarray*}$$

The intermediate K_x_MoS_2_ finally trended to form 1T^′^ MoS_2_.

### Wafer-scale epitaxial growth

**Figure 6.**
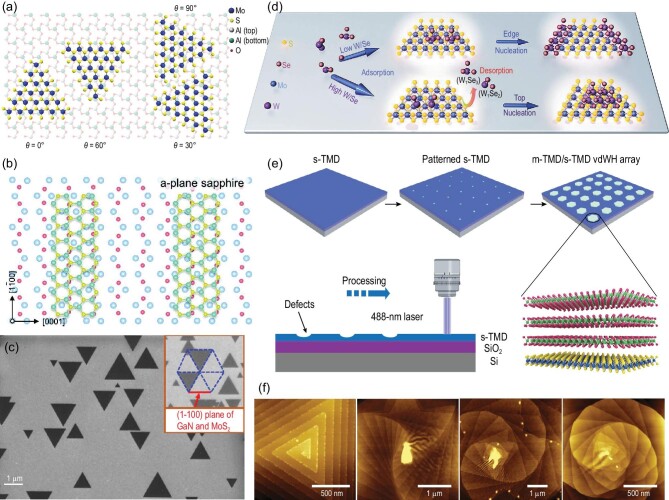
(a) Schematic drawing showing the top view of relative lattice orientations between monolayer MoS_2_ and *c*-plane sapphire [118]. Copyright 2015, American Chemical Society. (b) *a*-plane (1120) sapphire substrates and the structure of MoS_2_ grains grown on them [122]. Copyright 2020, WILEY-VCH Verlag GmbH & Co. KGaA, Weinheim. (c) Scanning electron microscopy of MoS_2_ monolayer triangles on GaN epitaxial crystal. The scale bar is 1 μm and is aligned with the (1100) plane of GaN. Orientation of the (1100) plane of hexagonal crystals of GaN and MoS_2_ is shown in the inset [124]. Copyright 2016, American Chemical Society. (d) Schematic view of the controllable growth process of lateral/vertical MoS_2_/WSe_2_ heterostructures with the adsorption of various active clusters W_1_Se_x_ [131]. Copyright 2019, WILEY-VCH Verlag GmbH & Co. KGaA, Weinheim. (e) Schematic of the growth process [132]. Copyright 2020, Springer Nature. (f) AFM images of representative WS_2_ supertwisted spirals with various twist angles grown around WO_x_ particles on SiO_2_/Si substrates [136]. Copyright 2020, American Association for the Advancement of Science.

For 2D materials to have realistic application, wafer-scale 2D films with few grain boundaries are essential. Inspired by the success in wafer-scale graphene growth via seamless stitching, much efforts have been devoted to growing wafer-scale 2D materials by aligning unidirectionally the grains on single-crystal substrates or amorphous SiO_2_ substrates. The symmetry of single-crystal substrates is a key factor in controlling the alignment behavior of 2D materials [115]. To obtain the unidirectional alignment of 2D materials, the symmetry group of substrates should be a subgroup of that of 2D materials. Recently, a wafer-scale single crystal of h-BN was synthesized successfully by CVD. Due to h-BN having a lower symmetry C_3_*_v_*, the suitable substrate for wafer-scale h-BN growth must have C_3_*_v_*, C_3_, σ_v_ or C_1_ symmetry. A Cu (110) vicinal surface, on which the presence of metal steps along the (211) direction led to a C_1_ symmetry, was applied in preparing unidirectional arrangement of the h-BN monolayer [116]. More than 99% unidirectional aligned h-BN grains were observed on the Cu (110) vicinal surface, whose zigzag edges were parallel to the Cu (211) step edge direction. This growth behavior breaks the equivalence of antiparallel h-BN domains, enabling unidirectional domain alignment. Recently, Chen *et al.* [117] obtained a similar result on Cu (111) with atomic steps, which can break the equivalence of 0° and 60° domain orientations. Similar to the symmetry of h-BN, most 2D TMDCs have 3-fold symmetry. The *c*-sapphire substrate, due to the close symmetry and compatible lattice constant with TMDCs, has been widely used to grow the TMDCs. Dumcenco *et al.* reported that there were three basic angles of 0°, 60° and 30° between monolayer MoS_2_ and *c*-sapphire, and Fig. 6a shows the top view of relative lattice orientations [118]. The grains were linked well in single-crystal MoS_2_ film, confirmed by local potential mapping along channels in FET. It was reported that an aligned WSe_2_ layer was acquired on the atomic step-terrace *c*-sapphire substrate. The atomic step-edge had two functions. The atomic steps provided active nucleation sites to generate aligned WSe_2_ nuclei. These periodic steps also provided a layer-over-layer overlapping to form aligned few-layer WSe_2_ flakes. This is different from the classical layer-by-layer mode in thin-film formation [119].

Furthermore, atomic layer h-BN with low symmetry C_3_*_v_* can also be used as the substrate to control the orientation of D_3_*_h_* subgroup symmetry of 2H-TMDCs. It has been seen that the relative rotation angles of the MoS_2_ domains were close to 0° on h-BN substrate while the as-grown MoS_2_ domains were arranged in a random orientation [120]. Yu *et al.* [121] developed a low-pressure CVD method to epitaxially grow monolayer MoS_2_ on the h-BN basal plane. In this process, only relative rotation angles of 0° or 60° between MoS_2_ and h-BN basal plane were observed. And the misaligned domains only occurred at the step-edge of h-BN. Domains with the same orientation were stitched and then formed single crystals, while domains with different orientations were stitched and caused mirror twin boundaries. When stitching the two types of domains, only mirror twin crystal existed in a continuous film. So, wafer-scale single crystal h-BN can be used as the substrate to grow the wafer-scale single crystal TMDC materials via seamless stitching.

Different symmetric substrates were also used to synthesize TMDCs. Ma *et al.* [122] obtained highly aligned MoS_2_ grains with an unusual rectangle shape on the *a*-plane of sapphire with 2-fold symmetry, as shown in Fig. 6b. Particularly, the MoS_2_ grains are similar to the results acquired on the *c*-plane of sapphire substrate, the morphology of which is triangle on *a*-plane sapphire with random orientation at 750°C growth temperature. In comparison, rectangle MoS_2_ grains with well-aligned orientation were observed at a temperature of over 900°C. Another 2-fold symmetric substrate m-quartz with anisotropic lattice match was applied to control the growth direction of monolayer WS_2_ [123]. A semiconductor GaN substrate has an IP lattice mismatch of <1% with MoS_2_ crystal, which can be used to orient the growth of monolayer MoS_2_. The match of thermal expansion coefficients between GaN and MoS_2_ allowed the synthesis of an unstrained MoS_2_ film, as shown in Fig. 6c [124]. It is notable that only polycrystalline TMDC films with randomly aligned domains have been synthesized on the amorphous SiO_2_ surface so far.

Another route to preparing wafer-scale single-crystal TMDCs is to form only one nucleus on a larger-area substrate and gradually grow it into a single crystal. However, the nucleation densities of TMDCs are difficult to control on polycrystalline or amorphous substrate. To address this issue, Ye *et al.* [125] very recently reported the seamless epitaxial growing of a 2D-confined single crystal via the solid-to-solid phase transition and recrystallization process. The seed layer 2H-MoTe_2_ was transferred on the polycrystalline 1T^′^-MoTe_2_, forming a vertical 2H/1T^′^ MoTe_2_ interface. Then, benefitting from the vertical 2H/1T^′^ MoTe_2_ interface, the 1T^′^-MoTe_2_ layer under the seed layer transformed into a 2H-MoTe_2_ single crystal, forming an IP 2H/1T^′^ MoTe_2_ interface. After that, the recrystallization and phase transition further occurred at the IP 2H/1T^′^-MoTe_2_ interface and extended outward. In such a process, the 2H/1T^′^-MoTe_2_ interface doubtlessly played a key factor. Therefore, other 2D materials (such as graphene and h-BN) could not take the place of 2H-MoTe_2_ to work as seeds for conducting the phase transition of the 1T^′^-MoTe_2_ at the vertical interface. This phase-transition method can also be used to synthesize other wafer-scale single crystal TMDCs with 1T/1T^′^ phase. Although the 1T or 1T^′^ phases of other TMDCs have higher energies, the salt-assisted method provides a useful tool for fabricating metal-phase TMDCs as discussed above. Thus, it is possible to synthesize wafer-scale TMDCs by combining the phase-engineering and salt-assisted methods together as discussed in the following.

As mentioned in the above section, a high growth rate is good for forming continuous monolayer films with millimeter-sized grains. The salt-assisted method enables the synthesis of  wafer-scale TMDCs at low temperature when used to generate less or one nucleus on a special substrate. For example, using solid soda-lime glass as the substrate directly, Yang *et al.* [126] successfully synthesized 6-inch uniform monolayer MoS_2_ via an elaborately designed feeding route of face-to-face metal precursor. In their experiments, it was found that the nucleation density can be effectively tuned by controlling the gap distance between Mo foil precursor and the glass substrate. Specifically, when the gap distance was ∼2 mm, the unsaturated S precursor tended to form irregularly shaped MoO_x_S_2−x_ crystals. When the gap distance was 10 mm, the nucleation density reduced dramatically, and the domain size was eventually up to 400 μm.

### Heterostructures based on CVD growth

Thanks to the dangling-bonds-free surface, vdW heterostructures can be fabricated by assembling 2D materials randomly. Besides the exfoliating-stacking technique, the heterostructure can be one-step assembled via CVD. In 2014, Ajayan *et al.* [127] reported WS_2_/MoS_2_ vertical heterostructures synthesized via CVD for the first time. After that, several similar heterostructures were also synthesized [97,98,128,129]. However, enlarging stacking area is stuck due to the random nucleation process. Meanwhile, the uncontrollable growth rates hamper the growth behavior of vertically stacked TMDC heterostructures. Fortunately, Zhang *et al.* [130] achieved a 100% stacking area of WS_2_/ReS_2_ heterostructures on Au (111) substrate. The adsorption energy of W atoms on Au (111) was relatively larger than that of Re atoms. Thus, the nucleation of W atoms was much easier than Re atoms. Once the WS_2_ is available, the Re atoms can nucleate on the WS_2_ surface to facilitate the growth of ReS_2_ due to the strong adsorption energy of Re on WS_2_ (001). To exactly control heterostructure growth, Li *et al.* [131] provided a detailed explanation about the nucleation and kinetics of TMDC vertical heterostructures growth, as shown in Fig. 6d. The nucleation on the surface of TMDCs was tied with the diffusion barrier of active clusters. There were two distinct active clusters W_1_Se_1_ and W_1_Se_3_ on the surface of MoS_2_. The W_1_Se_1_ with a higher diffusion barrier (≈1.2 eV) preferred to accumulate on the surface of MoS_2_, leading to the formation of the vertical stacking heterostructure. W_1_Se_3_ with lower diffuse barrier (≈0.4 eV) tended to diffuse to the edge of MoS_2_, forming a lateral heterostructure. In the experiment, the diffusion barrier of active clusters was associated with the ratio of metal to chalcogenide in the vapor precursors.

To overcome the difficulty of nucleation on 2D templates, a method of damaging the atoms of 2D templates was intentionally proposed [132]. In such cases, the exclusive defects with high energy could provide enough nucleation sites to grow vdW heterostructures. As shown in Fig. 6e, the bottom-layer 2D semiconductor TMDCs were grown by CVD first, and the periodic defect arrays were conducted on its surface, providing exclusive nucleation sites to site-specific growth of the top layer. Taking WSe_2_ as an example, after irradiation by focused laser, most W-terminations were exposed on the surface of WSe_2_ at the patterned sites due to the more volatile feature of Se. According to the calculation result of adsorption energy, top-layer VSe_2_ exactly preferred to form nucleation on W-terminated locations on WSe_2_. This general strategy was used to fabricate other TMDC heterostructure arrays, such as NiTe_2_/WSe_2_, VS_2_/WSe_2_ and VSe_2_/MoS_2_. It is worth noting that the growth temperature of top-layer metal TMDCs is precisely controlled to avoid the bottom layer decomposing, and the crystal size of the top layer can be controlled by adjusting the processing time.

In recent years, twisted 2D TMDCs have attracted tremendous research interest because of their unique properties that are essential in developing future electronic and optoelectronic devices. Apart from the ‘tear and stack’ technique, the twisted 2D TMDCs can also be directly grown by CVD. Liu *et al.* [133] showed that the twisted MoS_2_ bilayer with angles of 0°, 15° and 60° was grown on mica and fused silica as well as a SiO_2_/Si substrate through ambient pressure CVD. The key step to making the vertical layer-by-layer growth mode preferable is to suppress the nucleation rate at the initial stage of growth. The ratio of MoS_2_ bilayer to monolayer is determined by growth time. Bilayers with a yield as high as 30% can be achieved when performing 10 min growth under 700°C. In general, the yield of an AA-stacked bilayer of *θ* = 0° is the highest (∼85%), followed by an AB-stacked bilayer of *θ* = 60° (∼10%). A similar result of twisted bilayer MoS_2_ growth was also observed by Lin *et al.* [134]. For twisted WS_2_, random twisted WS_2_ bilayers with angles of 0°, 13°, 30°, 41°, 60° and 83° were found at high growth temperature (1100°C), while only AA- and AB-stacking bilayers were observed at a lower temperature (850°C) [135]. It is worth noting the latest work about the continuously twisted superstructures of WS_2_ and WSe_2_, where Zhao *et al.* [136] introduced non-Euclidean surfaces by dropping casting nanoparticles as protrusions on substrates, as shown in Fig. 6f.

## CONCLUSION AND OUTLOOK

Recent decades have witnessed the great development of 2D materials in low-power consuming transistors, high-gain photodetectors, ultra-sensitive gas sensors and flexible electronic devices due to their high mobility, atomic thickness and tunable bandgap. Many novel 2D materials with various interesting phenomena, including topology, piezoelectricity, ferroelectricity, magnetism and twisted heterostructure, have emerged in recent years, adding new vitality to the development of 2D materials [137]. The properties of emerging 2D materials can be easily modulated through the external electric field and magnetic field, due to the drastically weakened screening effect. The 2D piezoelectric, ferroelectric and magnetic materials, especially, provide a chance to break the limitations of the size effect and a promising platform for the realization of energy-efficient non-volatile memories, spintronics, logic devices, etc. However, substantial challenges for practical application still remain: (i) the stability of 2D piezoelectric, ferroelectric and magnetic flakes with atomic thickness under ambient conditions needs to be solved urgently in the future; (ii) the convinced mechanisms of spin-electron coupling and magnon in 2D system are still lacking—improving the understanding of these mechanisms will help us find ideal directions and suggestions for exploring novel 2D materials with specific functions through theoretical calculations; (iii) high *T_c_* magnetic materials need to be explored, although the *T_c_* can be improved to near 300 K by way of ionic gating and chemical stoichiometric ratio tuning.

Apart from the above aspects, an important factor for future industrial application of 2D materials may be the efficient fabrication of high-quality atomically 2D flakes on a large scale with low costs and simple crafts. For the top-down synthesis method, the large-area atomically 2D thin flakes with wafer scale have been obtained in both electrochemical intercalation exfoliation and metal-assisted mechanical exfoliation. These have new requirements for the preparation of 2D bulk single crystals, inspiring researchers to explore effective methods for the preparation of large-area, high-quality 2D bulk materials. Nevertheless, there are still some shackles that have not been shaken off. The cations introduced by cathodic electrochemical intercalation will be absorbed on the surface of the atomically 2D flakes and will then form stable ionic bonds, which is an obstacle with regard to studying the intrinsic properties of 2D materials and the assembly of devices. The inherent drawbacks of liquid-phase exfoliation, including agglomerations, the limited size of 2D flakes with arbitrary shapes and random distribution on substrates, also require more effort if they are to be resolved in the future. In addition, expensive metals will be hugely consumed in metal-assisted mechanical exfoliation, which is unfeasible for industrial preparation. Some inexpensive and friendly alternatives to 2D materials, such as organic polymers, will receive more attention when it comes to the controlled exfoliation of high-quality, large-area atomically 2D flakes in the future.

CVD is regarded as a proper candidate for large-scale fabrication of atomically 2D flakes with high productivity. However, it is still a big challenge to grow wafer-scale single crystal 2D materials via forming one nucleus on the substrate and developing it into a single crystal due to the high nucleation density. Until now, the utmost possibility to fabricate the large-area single-crystal monolayered 2D materials is to seamlessly stitch grains on special substrates. If the symmetry group of all 2D material contains the symmetry group of a substrate surface, 2D domains should show the same orientation during the CVD growth process. According to this principle, the larger-scale single-crystal h-BN monolayer was synthesized on a low-symmetry Cu (110) vicinal surface. This result indicates that TMDC materials can be synthesized on proper low symmetry substrate.

In summary, emerging 2D materials exhibit many interesting phenomena and potential for the fabrication of low consumption, minimized and integrated functional electronics. The preparation of wafer-scale 2D flakes has also made significant progress, displaying a prosperous situation. With the achievement of obtaining large-scale and high-quality samples, 2D materials will become indispensable as a part of electronic and optoelectronic technology and products in the post-Moore era.
